# Exploring the extended role of the community pharmacist in improving blood pressure control among hypertensive patients in a developing setting

**DOI:** 10.1186/s40545-017-0127-5

**Published:** 2017-12-21

**Authors:** Afia Frimpomaa Asare Marfo, Frances Thelma Owusu-Daaku

**Affiliations:** Faculty of Pharmacy and Pharmaceutical Sciences, Kumasi, Ghana

**Keywords:** Hypertension, Pharmacist, Pharmaceutical care, Blood pressure, Pharmacy

## Abstract

**Background:**

In most developing countries including Ghana, there is scant literature on the involvement of the community pharmacist in the care of patients with chronic conditions such as hypertension and blood pressure control. The objective of the study was to evaluate the effect of a pharmaceutical care model on blood pressure control and adherence among hypertensive patients.

**Methods:**

This was a quasi experimental design and the primary outcome measure was a change in systolic and diastolic blood pressure. One hundred and eighty hypertensive patients were recruited for the study: 90 in the intervention group and 90 in the control group. The intervention, consisting of health education, adherence counselling and medicine use review; was offered monthly for six months.

**Results:**

At baseline there was no significant difference in demographic and clinical characteristics between the intervention and control group. Pharmaceutical care issues identified among the intervention group during the 6 months period were non effectiveness of therapy (*n* = 23), experience of side effects (*n* = 20) and nonadherence to therapy (*n* = 40). The mean diastolic blood pressure difference between the intervention group and the control group was statistically significant (*p* = 0.001). The mean adherence difference between the two groups was also statistically significant at the end of the study. (p = 0.001).

**Conclusions:**

The pharmaceutical care intervention offered by the pharmacist led to the resolution of some pharmaceutical care issues, improvement in diastolic blood pressure and adherence among hypertensive patients. Guidelines and polices to streamline these services are needed if they are to be made available in community pharmacies in developing countries.

## Background

Hypertension is currently on the rise in most developing countries including Ghana [1]. The crude prevalence of hypertension in rural and urban Ghana ranges from 19.3% to 54.6% and it was among the first five commonest cause of outpatient morbidity in most regions in the country [2, 3]. The lack of awareness and inadequate treatment of hypertensive patients are factors contributing to the high burden of hypertension in Ghana [4]. The typical doctor’s review period for a Ghanaian hypertensive patient is every three months and yet blood pressure control is not adequate and patients lack information about their medicines and life style modifications [5]. There is therefore the need for services that would improve blood pressure control and prevent complications among patients with hypertension.

Community pharmacies in Ghana are privately owned and licensed by the Pharmacy Council with a mandate to promote rational use of medicines [6]. According to the Health Professionals Regulatory Bodies Act, a pharmacist can provide medical care as first aid during an accident and also provide treatment for illness of common occurrence when consultation with a medical practitioner or dentist is not reasonably practicable [6]. It has, however, been observed that community pharmacies in most low income settings are often underutilized and the standard of care and services tend to be suboptimal [7]. Currently, community pharmacists in Ghana do not normally follow up and monitor patients with chronic diseases as these often visit the pharmacies only for prescription refills.

Although studies on how the community pharmacist can assist hypertensive patients control their blood pressure have been conducted with largely positive outcomes in some developed countries such as England, United States of America and Canada, not much has been done in the developing setting [8–11]. Moreover the interventions studied and the number of times pharmaceutical care was offered to hypertensive patients by the community pharmacist varied within these studies and not all pharmaceutical care issues were reported. Duplicating similar services in Ghana would therefore require evaluating a pharmaceutical care intervention suitable for the Ghanaian community pharmacy. The information obtained would be essential for the purposes of policy initiation and planning with regard to the extended roles of community pharmacists in caring for patient with chronic diseases.

This study was carried out to evaluate the effect of a pharmaceutical care intervention on blood pressure control, adherence and adoption of healthy lifestyle practices among hypertensive patients. In addition we sought to determine the pharmaceutical care issues that are common among hypertensive patients and the level of satisfaction among hypertensive patients to whom the intervention was offered.

## Method

### Study design and setting

This non-randomised controlled study was conducted in five community pharmacies, in or near the Kumasi metropolis in the Ashanti region. The design involved the generation of two comparison groups because it was not feasible to randomise individuals into treatment and control groups due to lack of logistics. The intervention was offered in three community pharmacies (the intervention group), Charma situated at Aputugya, Kama at Bomso and Bandy at Adum. The control group comprised Marrita pharmacy, situated at Dompoase-Aprabo and Royal Avenue pharmacy at Manhyia. The major activities in these pharmacies included dispensing prescription and non-prescription drugs, treating illnesses of common occurrence, counselling and drug information dissemination.

Purposive sampling to select the five pharmacies was based on the following criteria. The pharmacist should be available during pharmacy opening hours, have at least one year experience in community pharmacy practice and should be easily available for training.

### Description of the intervention

The intervention offered was based on the pharmaceutical care model proposed by the American College of Clinical Pharmacists [12]. This model consists of three principal components; identifying actual and potential medication related problems, resolving actual medication related problems and preventing potential medication related problems. Drug therapy issues were categorized into four groups. These are indication, effectiveness, safety and adherence. Based on the pharmaceutical care model, the intervention offered by the community pharmacist was monthly medicines use review, health education and adherence counselling.

#### Medicine use review

The objective of the review was to identify and resolve drug related problems, focusing on improper drug selection, sub therapeutic doses, over dosage, adverse drug reactions, nonadherence to therapy, drug interactions and medicines use with no indication. In resolving drug therapy problems a care plan was developed via the SOAPO model [Subjective data, Objective data, Analysis, Plan and Outcome]. The subjective data was any signs and symptoms reported by the patient. The main objective data considered was the blood pressure and body mass index measurements. Based on the objective and subjective data; and medication history, an assessment of the medication related problem was made. The plan mainly consisted of recommended therapy, goals of therapy; counselling and monitoring. Patients and other health workers were involved in these plans. Patients were informed about all the necessary actions in the care plan and recommendations for medication regimen change; and elevated blood pressure was communicated to a doctor. In hypertensive patients without diabetes the targeted blood pressure was less than or equal to 140/90. In diabetic hypertensive patients the targeted blood pressure level was less than 130/80.

#### Health education

A concordant approach was used in educating the patient; thus patients’ views and suggestions was considered before conclusions were drawn. Patients were also educated on the common side effects of their antihypertensive medication, precautionary measures associated with taking their medicines, individualized healthy life style practices that would help in controlling their blood pressure and consequences of not adhering to their therapy. Life style practices discussed included physical activity, diet (in particular sodium reduction and a diet rich in fruits and vegetables and less fat), weight loss management, reducing alcohol intake and smoke cessation.

#### Adherence counselling

Two strategies were used in improving patient adherence. Dissemination of knowledge (making sure that the patient was aware of the name, dosage, side effects and purpose of their medicines) and simplifying dosage regimen.

### Training

A two day taught and practical training programme was given to the three pharmacists in the intervention group. The topics covered included pathophysiology, prevalence, risk factors, causes, diagnosis, and complication of hypertension. Goals of therapy, pharmacological methods available for managing hypertension, factors to be considered when choosing an appropriate therapy, and medicines available for the management of hypertension. Development of pharmaceutical care plans (SOAPO model), description of the pharmacist led hypertension pharmaceutical care service and procedures and protocols used in the project were also taught. Pharmacists in the control group were taught only the study procedures in the recruitment of subjects, and measurement of blood pressure and body mass index.

### Inclusion/exclusion criteria

Hypertensive patients diagnosed for at least six months with a review period of at least two months were included in the study. The only co-morbid condition included was diabetes. Other hypertensive patients with comorbid conditions such as heart failure, stroke, and ischaemic heart disease, renal and liver diseases were excluded from the study. Furthermore pregnant women, patients with no contact numbers and patients who would not be able to return to the pharmacy for a scheduled visit were excluded from the study.

### Sample size & sampling

The formula proposed by Charan & Bisawa, for calculating sample for size interventional studies [13, 14] yielded a sample of 180 hypertensive patients. Purposive sampling was used to select the first 180 patients who were within the inclusion criteria. Ninety patients were recruited into the control group: 45 patients from each of the two pharmacies; and ninety patients into the intervention Group. (IG): 30 from each of the three community pharmacies.

### Identification & recruitment

Eligible hypertensive patients were identified when they visited the pharmacy with a refill prescription that had a diagnosis of hypertension. The blood pressure was measured at the pharmacy with an automated Sphygmomanometer to ensure that about 50% of the total study sample in both the intervention and control group had blood pressure levels above 140/90. When recruited the purpose and design of the study were also explained to the patient by the pharmacist. Patients then appended their signature or thumb print to a consent form if they agreed to be part of the study.

### Outcome measures

The following outcomes were assessed at baseline and at the end of the study in both groups. However outcome 7 and 9 were assessed in only the intervention group (Table 1).

**Table 1:** Outcome Measures

	Outcome	Measure
1	Blood pressure (systolic and diastolic BP)	A validated automated blood pressure monitor (Omron M6) was used in measuring the blood pressure. Blood pressure was measured after the subject had rested for 5 min. Each blood pressure measurement recorded was based on an average of two blood pressure readings.
2	Body Mass Index (BMI)	BMI was determined with an equipment (Height And Weight Scale TZ 120) that measured the client’s weight and height. Then the formulae “weight in kg /Height in m ^2^”was used in computing the BMI.
3	Level of adherence to antihypertensive	Patient’s self-report using the 8 item Morisky scale
4	Alcoholic Status and frequency	Subject’s answer (Yes/No) to drinking alcohol The amount of alcohol consumed in a day was self-reported
5	Consumption of salty food	Yes/No to question “Do you eat salty food?
6	Frequency of exercise	Whether the subject exercised or not (Yes/No) Amount of aerobic physical activity (e.g., brisk walking, Skipping) undertaken within a week
7	Modifications in life style modifications	The number of hypertensive patients who reported adopting healthy life style practices at the end of the study
8	Pharmaceutical care issues	The number of pharmaceutical care issues identified and the action taken to resolve it.
9	Patient Satisfaction	Survey

### Data collection

Data were collected at baseline and then after 6 months for the control group. In the intervention group, the pharmacist initially interacted with the patient and offered the pharmaceutical care services described above. Information documented at baseline for both groups were: demographic characteristics, medication history (current medicines for the management of hypertension and other Over The Counter drugs and alternative medicines), life style practices (smoking status, frequency of exercise, alcohol use, consumption of salt), awareness of the name, dosage, frequency, side effect and purpose of antihypertensive prescribed and awareness of healthy lifestyle modification for managing blood pressure. Other information documented were blood pressure, body mass index measurement and level of adherence to antihypertensive medication. Additional information documented for the intervention group were pharmaceutical care issues and solutions offered. Follow up dates for subsequent visits were agreed with the patients and SMS messages were sent to remind patients of these dates. If patients failed to show up, a call was made to ascertain why the appointment was missed and a new follow up date was agreed. Patients with drug therapy related problems that needed change in therapy were referred to their normal review clinic.

During the intervention group’s monthly visits, the pharmacist had a 10–30 min face-to-face consultation with the patient. In the consultation the pharmacist measured the patients’ blood pressure and BMI. Identified drug related problems were discussed with the patient and a concordant solution sought. After the sixth month the same information documented at baseline was determined in the two groups.

For the patient satisfaction survey, a semi structured questionnaire was used to conduct a face to face interview among the 75 hypertensive patients in the intervention group after the sixth month. The questionnaire captured data on patient satisfaction with the medication use review service, health education and adherence counselling. Questions on client’s satisfaction with the overall services provided by the pharmacist and if pharmacist should be paid for offering the service were open ended questions. All other question where closed ended.

Pharmacists who offered the intervention were remunerated over the study period.

### Data analysis

Descriptive statistics in SPSS Version 16 were used to analyse baseline demographic and clinical data. Patients who did not exercise or patients who exercised less than 30 min per day, in less than 3 days were coded as ‘no exercise’. Participant who drank more than 2 drinks per day for men and more than 1 drink per day for women were considered as regular users of alcohol.

Adherence to antihypertensive medication was measured with the Morisky self-reported scale. A ‘yes’ answer attracted a score of Zero (0) and a ‘no’ answer a score of one. Patients who scored 8 were considered highly adherent, a score of 6- < 8 was classified as medium adherence and a score below 6 was classified as low adherence. The paired sample T test was used to compare means of end of study outcomes (SBP, DBP, adherence and BMI) within the IG and the CG. McNemar test was run to determine if there was any difference in the proportion of subjects who ate salty food, consumed alcohol and exercised pre and post intervention. The general linear model (repeated measures) analysis was run to compare the difference (SBP, DBP, adherence and BMI) between the two groups at baseline and after 6 months. Then the Wilks Lambda test was used to determine if there was any differences between the two groups. The Fishers exact test of association was employed to determine any association between the intervention offered and achievement of blood pressure goal. Cramer’s *V* test was used to determine the strength of the association; values of 0–0.30 were considered weak, 0.31–0.70 moderate, and 0.71–1.0 strong. In the analysis of data, all *p* values less than 0.05 were deemed significant.

## Results

One hundred and eighty hypertensive patients were recruited. Eight patients in the intervention group dropped out of the study because they moved out of the region while 6 patients were not reachable for subsequent follow-ups. Nineteen patients in the control group were unreachable for follow up after the 6th month. Participants who finally completed the study were thus 75 and 71 for the intervention and control groups respectively.

### Demographics characteristics of subjects at baseline

The mean age for the intervention group was 56.56 ± 9.197 whiles that for the control group was 53.45 ± 8.299. There was no significant difference in demographic characteristics of the two groups at baseline (Table 2).

**Table 2:** Baseline characteristics of Subjects

Demographic Characteristics		Intervention Group	Control Group	*p*-value
Sex n (%)	Male	33(44)	29 (40.8)	0.700
	Female	42(56)	42 (59.2)	
Age n (%)	36–45	12 (16.0)	15 (21.1)	0.166
	46–55	22 (29.3)	26 (36.6)	
	56–65	28 (37.3)	23 (32.4)	
	Above 65	13 (17.3)	7 (9.9)	
Educational Background n (%)	No education	10 (13.3)	8 (11.3)	0.497
	Basic	14 (18.7)	10 (14)	
	Middle School	17 (22.6)	19 (26.7)	
	Secondary	18 (24)	20 (28.2)	
	Tertiary	16 (21.3)	14 (19.7)	

### Drug therapy problems identified among the intervention group and solutions provided

Pharmaceutical care issues identified were related to effectiveness of therapy, side effects and adherence to therapy. Tables 3, 4 and 5, illustrate the various categories of pharmaceutical problems and actions taken to resolve them. The mean time spent in offering the pharmaceutical care to hypertensive patients was 14.88 mins ±3.78818 (Range10–32 min).

**Table 3:** Pharmaceutical issues related to effectiveness of therapy and action taken to resolve them

Pharmaceutical care issues	n (%)	Recommendations to clinician and action taken by clinician to resolve it (italicized)
Effectiveness of therapy (B)		
Patients whose blood pressure levels were above or equal to 160/100	15(20)	Dosage adjustments and/or add on therapy. ^a^ *Changed dosage of antihypertensive (n = 9)* ^a^ *Added an additional antihypertensive (n = 5)*
Aspirin prescribed when BP was not controlled.	5(6.67)	Aspirin be discontinued. ^a^ *Aspirin discontinued(n = 3)*
Atenolol and carvedilol prescribed as monotherapy for the management of hypertension.	2(2.60)	Beta blocker be changed to calcium channel blocker or a diuretic ^a^ *Beta blocker changed to a diuretic (n = 2)*
Furosemide prescribed for the management of hypertension	1(1.33)	Furosemide to be changed to bendrofluazide ^a^ *Furosemide substituted with bendrofluazide*

^a^Action taken by the physician on referral

**Table 4:** Pharmaceutical problem Related to side effects of medicine and Action Taken to resolve them

Safety (C)	n (%)	Recommendations and action taken to resolve it
Side effects of medicines		
Drowsiness and dizziness on taking methyldopa, nifedipine, lisinopril bendrofluazide	1 (1.33)	Advised patient to take lisinopril and nifedipine at night if sleep disruptions are minimal or to take medication with meals.
Blurred vision with hydrochlorothiazide	1(1.33)	Recommended to clinician that hydrochlorothiazide changed to calcium channel blocker. *Hydrochlorothiazide changed to Nifedipine*
Headache due to Nifedipine	8(10.67)	Advised patients: To take paracetamol for headache To take nifedipine at night Referred two patients with persistent headache to clinician. *Paracetamol prescribed for one and nifedipine substituted with amlodipine for other one.*
Sexual weakness with methyldopa	1(1.33)	Recommended patient to clinician for change of antihypertensive ^a^ *Methyldopa not changed*
Cough due to lisinopril	3 (4.00)	Recommended that Lisinopril should be changed to losartan. *Lisinopril changed to Bendrofluazide* *Lisinopril changed to amlodipine* *Lisinopril changed to losartan*
Frequent urination due to bendrofluazide	5(6.67)	Advised patients to always take bendrofluazide in the morning
Constipation with furosemide	1(1.33)	Advised patient to take in lots of fluids and referred to clinician for change in therapy. *Furosemide substituted with bendrofluazide*

^a^Action taken by the physician italicized

**Table 5:** Pharmaceutical problem Related to nonadherence of therapy and Action Taken to resolve them

Adherence (D)		
Nonadherence	n (%)	Recommendations and action taken to resolve it
Patient has not taken medication for the past two weeks because she feels blood pressure is low. She was given lisinopril 20 mg instead of 10 mg daily on her last visit to the hospital	1 (1.33).	Agreed with the patients on measures that would aid adherence to therapy, such as; • To always check blood pressure in a health facility and seek assistance when she feels her BP is low • Education on the need for continuous medication use.
Patient decided to double the dosage of Nifecard to 30 mg twice daily as she was not well	1(1.33)	Recommended that patient seeks advice from a health practitioner before adjustment on dosages is done.
Patient only takes medicines when symptoms such as headache or dizziness are felt.	11(14.6)	Agreed with patients on regular medication use and offered education on the advantage of continuous medicines administration.
Stopped taking antihypertensive and currently on herbal medicines.	5 (6.67)	Advised to see herbal medicine practitioner at the Kumasi South Hospital.
Noncompliance to doctor’s appointment	10(13.3)	Agreed with patient on measures that would ensure adherence to the doctor’s appointment These measures were; • The pharmacist/family member reminding patients of the appointment • Seeking financial help for transportation to the hospital • Prompt renewal of national health insurance Offering education on the importance of adhering to the doctor’s appointment.
Using antihypertensive with herbal medicine for fever	3 (4.0)	Advised patient not to take medicines together.
Patient has not taken medication because antihypertensive is finished and has not visited the hospital for a refill.	9(12.0)	Agreed with patient on measures that would ensure continuous administration of medication and ways to acquire a refill when medicines were finished. Such measures were: • Keeping the medicines pack and consulting a community pharmacist for few days refill • Visiting the hospital for a refill before medicines get finished. • Pharmacist/family member reminding patients of refill appointments • Education by pharmacist on the importance of continuous medicines administration.
Sub Total	40	

^a^Action taken by the physician on referral

### Outcome measures after 6 months follow up

There was an association between the intervention and the mean SBP: both groups showed a decrease in SBP at the end of the study. However the mean SBP difference between the two groups for the time period was not statistically significant. (F = 101.569, *p* = 0.697 partial η2 = 0.001). There was an association between the intervention and the mean DBP during the time period: the intervention group showed a decrease in DBP and the control group showed an increase in DBP at the end of the study (Table 6). The mean DBP difference comparing the two groups was significant. (F = 20.250, *p* = .000 partial η2 = .123) There was also a statistically significant association between the intervention offered and attainment of BP goal [OR, 2.509 95% CI, 1.683–3.739, *p* = 0.21] however the association was weak (=φ .177). There was an association between the intervention and adherence, the intervention group showing an increase in mean adherence score and the control group showing a decrease in adherence scores at the end of the study. The difference in the mean adherence scores between the two groups was statistically significant. [F = 218. 851, *p* = .000 partial η2 = 0.228].

**Table 6:** Clinical Characteristics of Subjects at baseline and end of Study

		Intervention group (IG)				Control Group (CG)			
Variables		Baseline	After 6 Months	Mean difference (95% CI)	*p*-Value	Baseline	After 6 months	Mean difference (95% CI)	*p*-Value
Blood Pressure	SBP Mean	152.3	143.4	9.28 (12.79 to 5.79)	0.000	147.2	145.8	1.34 (4.95 to −2.28)	0.463
	DBP Mean	87.6	78.5	9.040 (12.77 to 5.30)	0.000	90.7	91.2	−0.535 (1.77 to −2.84)	0.644
BMI	Mean	27.5	26.0	1.463 (2.33 to −0.59)	0.001	27.1	27.6	−0.098 (0.66 to −0.86)	0.798
Adherence	Mean	5.5	6.2	−0.693 (−0.08 to −1.30)	0.027	4.3	3.9	0.408 (0.765–0.052)	0.026
Smoking	n (%)	2 (2.7)	2 (2.7)		1.000	0 (0)	0(0)		1.102
Consumption of Salty foods	n (%)	28 (37.3)	15 (20.0)		0.011	19 (26.8)	16 (22.5)		0.508
Alcohol n (%)	Regular n (%)	8 (10.6)	2 (2.7)		0.000	9 (12.7)	3 (4.2)		.005
Exercise n (%)	n(%)	24 (32.0)	58 (77.3)		0.000	32 (45.1)	22 (31.0)		0.031

### Patient satisfaction with intervention

Patients were satisfied with the medicines use review and health education conducted by the pharmacists. As some patients put it. Seventy three percent of patients within the intervention group were most of the time satisfied with the service provided by the pharmacist (Table 7).

**Table 7:** Patient Satisfaction Survey

Variables	Most of the time	Sometimes	Very rarely	Never
I have to wait for a long time before my blood pressure is measured	4 (5.3)	7 (9.3)	50 (57.6)	14 (18.7)
I have to wait for a long time before my weight and height is taken	5 (6.7)	21 (28.0)	39 (52.0)	10 (13.3)
The pharmacist spends as much time with me as necessary	24 (32.0)	42 (56.0)	7 (9.3)	2 (2.7)
I have trouble understanding the education given to me	3 (4)	12 (16.0)	33 (44.0)	27 (36.0)
The pharmacy staff are friendly	45 (60)	25 (33.3)	5 (6.7)	0 (0.0)
Follow up appointments are helpful	32 (42.7)	34 (45.3)	6 (8.0)	3 (4.0)
The advice given by the pharmacist helped me in taking my medicines	48 (64.0)	23 (30.7)	3 (4.0)	1 (1.3)
I am very satisfied with the pharmacy service received	55 (73.3)	18 (24.0)	2 (2.7)	0 (0.0)

*The pharmacist explains the possible side effects that my medication may cause and also the service has helped me in taking my medicines. P1*

*The pharmacist makes sure that I understand how to take my medication and if I have a question about my medicines the pharmacist is always available to help me. P2*

*I am very satisfied with the service offered by the pharmacist, the advice given by the pharmacist has helped me change some unhealthy life style practices. P3*

However there was some mixed opinions on whether the pharmacists should be paid for offering the service.

*The pharmacist should be paid for offering such a service and this service should be provided to all hypertensive patient P4*

*Although the service provided by the pharmacist has helped me in taking my medicines, I cannot regularly pay for such a service. P5*

## Discussion

### Pharmaceutical care issues identified among the pharmacist intervention group and solutions provided

The common care issues identified was nonadherence to antihypertensive therapy. These were missed doses, lack of medicines and missed doctor’s appointments. Some studies among hypertensive patients have also reported nonadherence as a major care issue commonly encountered [15]. Another category of pharmaceutical problems reported was experiencing side effects of the antihypertensive; an issue often associated with chronic diseases [16]. Solutions provided were mostly changes in therapy because if patients continued experiencing side effects it was likely they would not take their medicines.

Low dose aspirin is an antiplatelet and its long term use is associated with major gastrointestinal and extracranial bleeding [17]. Patients whose blood pressure are not optimised are at risk of complications such as stroke and this could be haemorrhagic if patients are on low dose aspirin. Therefore low dose aspirin is recommended for the primary prevention of cardiovascular events in hypertensive patients aged over 50 years with a baseline CVD risk level > 20% and whose blood pressure has been controlled to an audit standard of 150/90 [18]. However some subjects were still on dispersible aspirin although their blood pressure was not controlled.

Another drug therapy problem encountered was the use of beta blockers as monotherapy in the management of hypertension. Beta blockers are often recommended as add on therapy when drugs such as diuretics and calcium channel blockers have not worked [19, 20]. Compared with placebo beta blockers are effective in reducing the blood pressure, however the blood pressure lowering efficacy of beta blockers are suboptimal compared to other antihypertensive. Furthermore there is evidence suggesting that beta blockers do not reduce mortality [21].

### Outcome measures after 6 months

The intervention studied led to a statistically significant mean DBP difference between the IG and the CG and there was an association between the intervention offered and achievement of BP Goal. Thus, subjects who were offered the intervention were more likely to achieve BP Goal than those who were not. A similar intervention (medicines review, adherence and counselling) for 118 hypertensive patients in a randomized study in Canada caused a significant reduction in SBP in the intervention group compared to placebo. The difference in the number of subjects who had their blood pressure controlled was also statistically significant. The average age of participants, 58 years, was similar to that in our study [22]. Achievement of BP control is essential as there is evidence that suggests that patients whose blood pressure is controlled to less than 140/90 often have a low incidence of cardiovascular complications such as stroke, myocardial infarction and death [19].

The mean adherence difference between the IG and the CG was also statistically significant and there was a moderate association between the intervention offered and adherence. Thus subjects who were offered the intervention were more likely to adhere to treatment than those who were not. Some studies on pharmacist intervention on adherence have shown similar or conflicting results. There was a significant difference in adherence level in the intervention and control groups in randomised studies in Thailand and United Kingdom [8, 10]. However another randomized controlled study showed no significant difference in adherence between the intervention and control groups [9].

### Implications of findings on practice

At baseline adherence levels of subjects in both groups was quite low. It is therefore essential that measures and polices are established to achieve high adherence levels among hypertensive patients. In most clinical settings in Ghana hospital folders lack documentation of adherence to therapy for patients with chronic diseases. Ideally adherence to medication should be assessed in the consultation room during each review visit before decisions on medicines are taken. In addition, adherence could also be assessed by the pharmacist before medicines are dispensed. This practice could enable these health care personnel to identify and address any adherence concerns of the patient.

The Ghanaian community pharmacist should be encouraged to monitor and offer similar interventions to patients with hypertension who come to refill their medications; as this can improve blood pressure control and adherence and enhance the public image of the pharmacy profession in the country. For such pharmaceutical services to be effectively rolled out in Ghanaian community pharmacies it is very important for some laws and policies to be updated. For instance the Ghana Pharmacy Act 489, (1994) which has been repealed and subsumed by the Health Professions Regulatory Bodies Act 867, is silent on the extended responsibilities of the community pharmacists for patients with chronic diseases [5]. The Pharmacy Council, Ghana in conjunction with the Community Pharmacy Pharmacist Association (CPPA) of Pharmaceutical Society of Ghana (PSGH) could determine policy guidelines on care services the community pharmacist can provide. However there is the need to explore measure to reduce the number of patients lost to follow up because pharmacy owners might not want to invest their time in offering such a service if clients are going to disappear.

Offering the intervention described in this study, is time consuming. The mean time spent with subjects was fourteen minutes and several phone calls and SMS were made in an attempt to remind them of follow up dates. It is therefore essential that the pharmacist is offered some remuneration as was done in the study and also there is the need to explore the cost effectiveness of the service. Over half of hypertensive patients agreed that the pharmacist should be paid for rendering such services and should offer it to all hypertensive patients. In the United Kingdom the community pharmacist is mandated to offer so many pharmaceutical services apart from their primary role and are paid a commission by the National Health Service (NHS) for each care service they offer [23].

### Limitations of the study

Some patients in the Control Group (CG) were mostly lost to follow up, probably because the first follow up occurred six months after the first encounter. Another reason why follow up appointments were missed was that in some instances the telecommunication coverage was poor, hence some clients were unreachable. The study also assumed that all patients who liked salty food consumed salty food. Furthermore a quasi-experiment design was chosen for the evaluation of the pharmaceutical care of hypertensive patients instead of a randomised controlled study which is the gold standard due to the lack of documentation of clients medical and medication history in the community pharmacy.

## Conclusions

It is feasible for Ghanaian community pharmacists to extend their services and continually offer pharmaceutical care (conducting medication reviews, assessing adherence and counselling) to patients with hypertension. This could lead to the identification and resolution of drug related problems and improvement in blood pressure control and adherence. However, for community pharmacies to effectively and continually offer these services, national policies would have to be enacted to provide some remuneration for the service. Community pharmacists in low income countries with increasing low levels of blood pressure control could adopt such an intervention. However, it is important that they consider their pharmacist staffing level and pharmacy setting. This is because this service requires the continuous presence of a pharmacist and space for offering pharmaceutical care to ensure privacy.

## Abbreviations

BMI: Body Mass Index
BP: Blood Pressure
CG: Control Group
CPPA: Community Pharmacists Association
CVD: Cardiovascular Diseases
DBP: Diastolic Blood Pressure
IG: Intervention Group
OR: Odd Ratio
P1-P5: Patients
PSGH: Pharmaceutical Society of Ghana
SBP: Systolic Blood Pressure
SOAPO: Subjective, Objective, Analysis, Plan and Outcome
SPSS: Statistical Package for social sciences
